# Individual-level factors attributable to urban-rural disparity in mortality among older adults in China

**DOI:** 10.1186/s12889-020-09574-9

**Published:** 2020-09-29

**Authors:** Yuan Zhao, Xin Xu, Matthew E. Dupre, Qianqian Xie, Li Qiu, Danan Gu

**Affiliations:** 1 Ginling College & School of Geographic Sciences, Nanjing Normal University, Nanjing, China; 2 International Center on Aging and Health & School of Geographic Sciences, Nanjing Normal University, Nanjing, China; 3 Department of Population Health Sciences & Department of Sociology, Duke University, Durham, NC USA; 4 School of Geographic Sciences, Nanjing Normal University, Nanjing, China; 5 Independent Researcher, New York, NY USA; 6 Independent Researcher, New York, NY USA

**Keywords:** Urban-rural disparity, Mortality, Older adults, Oldest-old, China, CLHLS

## Abstract

**Background:**

Urban-rural disparity in mortality at older ages is well documented in China. However, surprisingly few studies have systemically investigated factors that contribute to such disparity. This study examined the extent to which individual-level socioeconomic conditions, family/social support, health behaviors, and baseline health status contributed to the urban-rural difference in mortality among older adults in China.

**Methods:**

This research used the five waves of the Chinese Longitudinal Healthy Longevity Survey from 2002 to 2014, a nationally representative sample of older adults aged 65 years or older in China (*n* = 28,235). A series of hazard regression models by gender and age group examined the association between urban-rural residence and mortality and how this association was modified by a wide range of individual-level factors.

**Results:**

Older adults in urban areas had 11% (relative hazard ratio (HR) = 0.89, *p* < 0.01) lower risks of mortality than their rural counterparts when only demographic factors were taken into account. Further adjustments for family/social support, health behaviors, and health-related factors individually or jointly had a limited influence on the mortality differential between urban and rural older adults (HRs = 0.89–0.92, *p* < 0.05 to *p* < 0.01). However, we found no urban-rural difference in mortality (HR = 0.97, *p* > 0.10) after adjusting for individual socioeconomic factors. Similar results were found in women and men, and among the young-old and the oldest-old populations.

**Conclusions:**

The urban-rural disparity in mortality among older adults in China was largely attributable to differences in individual socioeconomic resources (i.e., education, income, and access to healthcare) regardless of gender and age group.

## Background

Urban-rural disparity in health has been shown to vary across time and place [1, 2]. Historically, urban areas have had higher rates of infectious disease and mortality at the early stages of industrialization due to poor sanitation, hazardous working environments, and high population density—leading to a so-called urban mortality “penalty” [3]. However, today’s urban areas usually have lower rates of mortality compared with rural areas due to improvements in infrastructure, better public health and medical systems, and overall advances in socioeconomic development [4–7]. These macro-level and institutional factors, along with a number of individual-level factors such as socioeconomic status [8], lifestyle and nutrition [9], and social networks [10], have been attributed to urban-rural disparity in mortality.

In contemporary China, mortality has been documented to be significantly lower in urban areas than in rural areas. According to the National Bureau of Statistics of China (NBSC), death rates at all ages have been lower for urban residents than for rural residents in the last four censuses [11–14]. Several studies based on national survey data have also showed excessive mortality among rural older adults for deaths among all causes [8, 15–19]. Other localized studies have further shown urban-rural disparity in colorectal cancer mortality and dementia-free life expectancy [20, 21]. In the last two decades, however, residents in urban areas have been shown to exhibit higher rates of physical inactivity, unhealthy eating, obesity, and hypertension than residents in rural areas [22–24].

Studies have further shown that improvements in healthcare services, socioeconomic resources, and infrastructure (e.g., transportation and safe drinking-water) in urban areas may partly explain the urban-rural disparity in mortality in contemporary China [4, 5, 8, 15, 25]. Although China has witnessed a rapid urbanization since the 1990s [26], the urban-rural disparity is still persistent in the healthcare system, pension system, and ultimately in health outcomes and mortality [7, 8, 18, 19]. Thus, identifying factors contributing to the mortality disparity between urban and rural older adults could have important implications for China to reduce the “health inequality” and to achieve good health and well-being for all in Goal 3 and reduced inequalities in Goal 10 of the Sustainability Development Goals (SDGs) set by the United Nations [27].

A notable limitation of the existing research is that although many studies have documented urban-rural disparity in mortality, most of them have not systematically examined which factors are the primary contributors to such mortality disparity [8, 17]. One exception is a study by Zimmer et al. that examined the link between neighborhood and individual socioeconomic characteristic and mortality among adults aged 50+ in China [8]. However, this analysis did not take into account other factors, such as social support, health behaviors, and baseline health status—which have been shown to influence risk of mortality among older adults in China, nor it examined the disparity by age group and gender [17]. Another limitation of the existing literature is that most studies have not focused on the factors associated with urban-rural disparity in mortality at the oldest-old ages and by gender. It is unclear what kind of individual-level factors are dominants in causing the urban-rural mortality disparity, and which process may underlie the urban-rural disparity in mortality in China at older ages, especially at the oldest-old ages.

Numerous frameworks have been applied to examine factors associated with health disparities (including urban-rural health disparity), of which the biopsychosocial (BPS) model of health is one of the most commonly used and probably the most appropriate framework in examining factors associated with health disparity [28]. The framework classifies factors associated with health/mortality disparities into biological factors, psychological factors, and social and ecological/environmental/contextual factors. Under this framework, two most widely recognized theories emerged. The cultural and behavioral theory suggests that differences in behaviors such as smoking, drinking, diet, and physical activities and differences in cultural norms that enhance or suppress such behaviors are the root causes of health disparity. The materialism and structuralism theory argues that it is disparities in socioeconomic environment such as income, wealth, power, opportunities, social capital, and institutions that lead to differences in health outcomes [29, 30].

Along with this line of the materialism and structuralism theory, three hypotheses are relevant in explaining whether differences in socioeconomic resources continues to lead to health/mortality disparities at older ages: the cumulative dis/advantage theory, the age-as-leveler theory, and the persistent inequality theory [31–35]. The cumulative dis/advantage argument suggests that differences in individual-level status and resources produce large and increasing disparities in health in later life. Alternatively, the age-as-leveler theory suggests that health/mortality disparities are largest in mid-life and largely diminish with advancing age—often attributable to mortality selection. The persistent inequality hypothesis argues that health disparities remain largely unchanged over the life course. All these three hypotheses have empirical support.

Guided by the PBS framework and aforementioned theories, the purpose of this study is threefold. First, it aims to examine urban-rural disparity in mortality at young-old (aged 65–79) and oldest-old (aged 80+) ages by gender in mainland China. Second, it aims to examine whether and to what extent socioeconomic conditions, family/social support, health behaviors, and baseline health status contribute to urban-rural disparity in mortality. Third, it aims to assess whether the associations differ by age group and by gender. The implications of the results are discussed in the context of population aging and geographical differences in mortality at older ages.

## Methods

### Study population

The data used in this study were from the Chinese Longitudinal Healthy Longevity Survey (CLHLS). The CLHLS is an ongoing nationwide survey staring in 1998. So far seven follow-up waves have been conducted in 2000, 2002, 2005, 2008/2011 and 2008/2009 (hereafter as 2008), 2011/2012 (hereafter as 2011), 2014, and 2018/2019. We did not include the first two waves of the CLHLS (1998 and 2000) and the last wave because the first two waves did not include adults aged 65–79 and the latest wave was not available when this research was undertaken.

The CLHLS was conducted in a randomly selected half of the counties/cities in 22 of 31 provinces in mainland China. Nine other provinces were not included in the sampling frame of the CLHLS because of concerns of age exaggeration at advanced ages. Overall, these nine provinces had much higher proportions of ethnic minorities in their population than the 22 sampled provinces. After 2008, Chenmai County in Hainan Province, one of the nine provinces, was included in the CLHLS as the accuracy of age-reporting was similar to that in the other 22 provinces. The share of the total population in these 23 provinces in the 2010 census was about 90% of the entire China (or approximately 89% if Hainan Province is excluded) [7].

The CLHLS aimed to interview all centenarians in the sampled counties/cities. For every three centenarians, four nonagenarians (aged 90–99), four octogenarians (aged 80–99), and five older adults (aged 65–79) were interviewed in nearby counties/cities. The list of names used for sampling was obtained from the household registration system at local public security agencies and/or residential committees. The selection of every sampled respondent was processed with a predestinated code for age and gender; so that the number of sampled persons of the entire survey at each age and each gender would be similar. In other words, the CLHLS oversampled older adults in their 80s, 90s, 100s, and older men to account for large attritions in follow-up waves due to high mortality. The response rates in these waves were approximately 88–90% [36].

For each sampled individual, the CLHLS collected baseline data on demographic background, socioeconomic conditions, family and social relationships, utilization of healthcare services, personality, birth history, sibling history, and health conditions—including physical and cognitive functioning and disease diagnoses. At follow-up interviews, in addition to several newly added questions, all aforementioned data were re-collected for those who were still alive and agreed to continue participating in the survey. For deceased persons, basic information prior to death was collected from their next-of-kin (such as urban-rural residence, marital status, living arrangements, physical function, disease conditions, healthcare utilization, place of death, and quality of death). Further details of the CLHLS sampling procedures and assessments of data quality are provided elsewhere [36, 37].

For the purpose of more robust estimates, we followed an approach used by prior studies and pooled the five waves of CLHLS in the analysis [38–40]. From 2002 to 2011, there were 33,512 respondents who contributed 57,285 observations to the CLHLS datasets. Out of these respondents, 783 (2.3%) survived to 2014, 8179 (24.4%) had 2+ interviews but were lost to follow-up, 19,273 (57.5%) died between 2002 and 2014, and 5277 (15.7%) had only one interview and were subsequently lost to follow-up. In the analysis, we excluded 5277 respondents because their survival status in any survey interval was unknown. Respondents who had 2+ interviews yet were lost to follow-up after their second interviews were considered censored observations in the analyses. The final valid analytic sample size was 28,235 individuals who were recruited from 2002 to 2011 and exposed to mortality from 2002 to 2014.

### Measurement

#### Urban-rural residence

The urban-rural residence information was collected at each wave of the CLHLS. We classified the urban-rural residence in a way consistent with the one defined by the National Bureau of Statistics of China [13, 41].

#### Mortality

All-cause mortality, the key variable of the present study, was measured by the length of exposure to death (duration) and the survival status at the time of the 2014 survey (event). The length of exposure was measured in number of days. The calculation for the length of exposure consisted of three parts: one for the deceased persons who died in 2002–2014, the second part for survivors at the time of 2014 survey, and the third part for those who were lost to follow-up. For the first part, the number of days was calculated from the date of the first CLHLS interview in 2002–2011 to the date of death. For the second part, the number of days was calculated from the date of the first interview in 2002–2011 to the date of the 2014 interview. For the last group, the number of exposure days was calculated from their first interview in 2002–2008 to their last interviews with their known survival status; afterwards they were excluded from the analysis since there were no observations for them. Dates of death were mostly from official death certificates. In some cases, they were from the next-of-kin and local residential committees. From 2002 to 2014, approximately 70% of respondents died (31.8% in the weighted data); and about 18.4% of respondents (24.8% weighted) were lost to follow-up. Urban respondents were 80% more likely to be untraced than rural respondents; primarily because of changes in residential address and/or relocation resulting from rapid urbanization [4]. The mortality data in the CLHLS is reported to be of high quality, as documented elsewhere [36].

#### Factors associated with the urban-rural disparity in mortality

Following the BPS framework and previous research in the field of health disparities, we examined a wide range of factors associated with urban-rural disparity in mortality [8, 17, 40, 42, 43]. Demographic characteristics included chronological age (in years), gender, and ethnicity (Han vs. non-Han). Socioeconomic factors consisted of number of years of formal schooling (no, 1–6, and 7+), economic independence (having a retirement wage/pension and/or own earnings vs. no), primary lifetime occupation (white collar occupation vs. other), family economic conditions (good vs. not good), and adequate access to healthcare (yes vs. no). Family/social support included marital status (currently married vs. no), proximity to children (close vs. not close; defined as either having a co-resident child or having a child living in the same neighborhood), and the first person to talk with when needing support (spouse, children/relatives, friends, social workers/housekeepers, and nobody). Health behaviors included three variables: smoking status (currently smoking yes vs. no), ever engaged in physical labor in the past (yes vs. no), and the participation in hobbies/leisure activities. Six hobbies/leisure activities were considered, which included reading books/newspapers or watching TV/listening to radio, doing housework, raising domestic animals or poultry or pet, gardening, and any other personal outdoor activities (e.g., fishing, walking, jogging, or exercise). Each activity was measured on a five-point Likert scale (from never to almost daily) and the scores were summed. Following previous research, respondents were grouped into three levels of participation: low level (never involved in these activities), high level (involved 1–7 times per week in at least one activity), and intermediate level (the remaining respondents) [40].

Health conditions included physical functioning, cognitive functioning, and chronic diseases. Physical function was defined by disability in activities of daily living (ADL) and instrumental ADL (IADL). The CLHLS collected data on six activities related to ADL: (a) bathing, (b) dressing, (c) indoor transferring, (d) toileting, (e) eating, and (f) continence [18]. Each item had three response categories: “able to do without help,” “need some help,” and “need full help.” We classified the respondents as ADL disabled (coded as 1) if they reported needing any help in performing any of the six items; otherwise we classified them as ADL not disabled (coded as 0). IADL disability items included eight self-reported activities: (a) shopping, (b) lifting a 5-kg bag, (c) walking one kilometer, (d) washing clothes, (e) cooking, (f) visiting neighbors, (g) taking public transportation, and (h) crouching and standing up three times. These IADL items in the CLHLS were adopted from the Nagi scale [37]. The classification of IADL disability was similar to that used for ADL disability. Respondents were classified as IADL disabled (coded as 1) if they reported needing any help in performing any of the eight items.

Cognitive function was measured using the Mini-mental State Examination (MMSE) that included six domains of cognition— orientation, reaction, calculation, short-term memory, naming, and language—with a total score of 30. The MMSE scale was adopted from the Folstein MMSE scale [44]. Respondents were classified as cognitively impaired if their MMSE score was below 24 [44]. Given the low level of educational attainment among most older adults in China, an alternative criterion (e.g., score of 18) for those with no education was applied to test the sensitivity of different cut-points for defining cognitive impairment (results available upon request); we obtained similar results to those presented here.

Chronic diseases were dichotomized into having 1+ self-reported disease condition versus none. The number of comorbidity condition included more than 25 health conditions such as hypertension, diabetes, chronic obstructive pulmonary disease, cardiovascular diseases, and cancers. According to previous study, approximately 95% of the conditions were self-reported as diagnosed by a doctor [37].

### Analytical strategy

We used a series of exponential hazard regression models to examine how different sets of other factors influenced the association between urban-rural residence and mortality. Seven models were evaluated. Model I included demographic background and survey year. Model II added socioeconomic factors to Model I. Model III added family/social support to Model I. Model IV added health behaviors to Model I and Model V added health conditions to Model I. Model VI included variables in Models III to Model V. Finally, Model VII included all study variables. Multicollinearity was assessed among all variables and did not pose a problem (all variance inflation factors were less than 3).

In the analytical sample, all variables had less than 2% missing values. We used their means to impute missing values for continuous variables and used their modals to impute missing values for categorical and dichotomous variables. A multiple imputation approach also was assessed and the results were almost identical to those based on the mean/modal imputations. Therefore, we only presented the results from the latter imputation method. Finally, the analyses controlled for CLHLS survey year to account for possible differences in mortality risks over time. Sampling weights were used in the hazard models to account for the study design of the CLHLS. Although urban respondents were more likely than their rural counterparts to be lost to follow-up, the urban-rural disparity in mortality was consistent when imputing survival information for those who were lost to follow-up (see Additional file 1). All analyses were performed using Stata version 15.0.

## Results

Table 1 presents the weighted distributions of the study variables. Overall, the demographic profiles of urban and rural older adults were similar. The proportion of respondents who died over the 2002–2014 period was relatively lower among urban older adults than that among rural older adults. Compared to the rural sample, urban older adults had much higher a socioeconomic status (SES) and were more likely to be married; however, they had less close proximity to their children compared rural older adults. Urban older adults were more likely to engage in leisure activities and had less IADL and cognitive impairments than their rural counterparts. However, older adults in urban areas had lower ADL function and higher rates of chronic disease than older adults in rural areas.

**Table 1:** Weighted Distributions of the Study Sample, CLHLS, 2002–2014

	Total	Rural	Urban		Total	Rural	Urban
Total sample #	28,235	17,170	11,046	**Family/Social Support (continued)**			
				First talking-to-person when needed			
Death in 2002–2014^a^	31.8	33.4	28.5***	Spouse	49.5	48.0	52.9***
**Urban-rural residence**				Child/relative	25.0	25.3	24.3***
Rural	32.0	100.0	–	Friend	22.6	23.7	20.0***
Urban	68.0	–	100.0	Social worker/housekeeper	0.6	0.4	0.9***
**Demographic Background**				Nobody	2.4	2.4	1.9***
Mean Age	71.9	72.0	71.7***	**Health Behaviors**			
Sex				Level of leisure activity			
Women	50.4	50.2	50.9	Low	8.7	9.4	7.1***
Men	49.6	49.8	49.1	Intermediate	51.2	54.2	44.5***
Ethnicity				High	40.2	36.4	48.4***
Non-Han	6.9	7.8	4.8***	Currently smoking			
Han	93.1	92.2	95.2***	No	72.8	72.4	73.7
**Socioeconomic Factors**				Yes	27.2	27.6	26.3
Years of schooling				Ever engaged in physical labor			
0	46.5	52.1	34.6***	No	15.6	8.9	29.9***
1–6	38.3	38.2	38.4***	Yes	84.4	91.1	70.1***
7+	15.2	9.7	27.0***	**Health Conditions**			
Economic independence				IADL disabled			
No	47.9	55.3	32.2***	No	68.8	67.5	71.5***
Yes	52.1	44.7	67.8***	Yes	31.2	32.5	28.5***
White-collar occupation				ADL disabled			
No	87.0	92.7	74.7***	No	93.8	94.3	92.8**
Yes	13.0	7.3	25.3***	Yes	6.2	5.7	7.2**
Family economic condition				Cognitively impaired			
Not good	84.3	85.9	81.0***	No	88.0	86.7	91.0***
Good	15.7	14.1	19.0***	Yes	12.0	13.3	9.0***
Got adequate access to healthcare				Has 1+ chronic disease			
No	7.7	9.1	4.7***	No	42.1	65.4	35.0***
Yes	92.3	90.9	95.3***	Yes	57.9	54.6	65.0***
**Family/Social Support**				**Survey Year**			
Currently married				2002	48.0	48.1	47.9***
No	34.8	35.6	33.0*	2005	18.0	15.8	22.6***
Yes	65.2	64.4	67.0*	2008	24.1	23.3	25.7***
Close proximity to children				2011	9.9	12.8	3.8***
No	15.7	12.0	23.5***				
Yes	84.3	88.0	76.5***				

Note: (1) Values are reported as weighted percentages or means with the exception for the total sample size. Urban and rural percentages were estimated separately; and percentages were estimated from the total sample size

(2) ^a^The unweighted percentage of deceased persons from 2002 to 2014 was 69.4% for rural areas and 66.4% for urban areas (68.3% combined)

(3) Statistical Tests (T-test for means, Chi-square tests for categorical variables) between urban and rural groups. *p < 0.05, **p < 0.01, ***p < 0.001

Table 2 presents the adjusted hazard ratios (HR) of mortality for urban versus rural older adults. Model I shows that urban older adults had about 11% lower risks of mortality (HR = 0.89; *p* < 0.01) than rural older adults after including demographic background and survey year. However, we found no urban-rural difference in mortality after including socioeconomic factors in Model II. Inclusion of family/social support (HR = 0.89; *p* < 0.01), health behaviors (HR = 0.91; *p* < 0.01), and health-related factors (HR = 0.90; *p* < 0.01) had a limited influence on the mortality differential between urban and rural older adults. Even when all three sets of factors were included in Model VI, urban older adults still had significantly lower risk of mortality (HR = 0.92; *p* < 0.05) than rural older adults—suggesting that these factors had limited power in explaining the urban-rural difference in mortality. Finally, there was no significant difference in mortality when all factors were included in Model VII.

**Table 2:** Adjusted Hazard Ratios of Mortality for Urban-Rural Residence, CLHLS 2002–2014

	Model I	Model II	Model III	Model IV	Model V	Model VI	Model VII
Urban (rural)	0.89**	0.97	0.89**	0.91**	0.90**	0.92*	0.96
**Demographic Background**							
Age	1.10***	1.09***	1.09***	1.09***	1.08***	1.07***	1.07***
Male	1.31***	1.45***	1.37***	1.30***	1.45***	1.45***	1.51***
Han (non-Han)	0.93	0.94	0.94	0.91	0.87*	0.88*	0.89*
**Socioeconomic Factors**							
1–6 years of schooling (0)		0.93+					0.98
7+ years of schooling (0)		0.82**					0.90+
Economic independence (no)		0.75***					0.81***
White collar occupation (no)		1.11*					1.07
Good family economic condition (no)		0.97					1.01
Adequate access to healthcare (no)		0.80***					0.91+
**Family/Social Support**							
Currently married (no)			0.88**			0.91+	0.94
Close proximity to children (no)			1.02			1.01	1.00
Primary support, child/relative (spouse)			1.05			1.05	1.04
Primary support, friend (spouse)			0.98			1.01	0.99
Primary support, other (spouse)			1.40+			1.14	1.15
Primary support, nobody (spouse)			1.45***			1.23**	1.22**
**Health Behaviors**							
Leisure activity level, intermediate (low)				0.59***		0.71***	0.73***
Leisure activity level, high (low)				0.47***		0.61***	0.64***
Currently smoking (no)				1.07		1.08+	1.08+
Ever engaged in physical labor (no)				0.98		1.00	0.96
**Health Conditions**							
IADL disabled (no)					1.40***	1.33***	1.30***
ADL disabled (no)					1.52***	1.39***	1.39***
Cognitively impaired (no)					1.36***	1.26***	1.25***
Has 1+ chronic disease (no)					1.09*	1.10**	1.10**
**Survey Year**							
Wave 2005 (2002)	0.82***	0.84***	0.83***	0.81***	0.84***	0.83***	0.84***
Wave 2008 (2002)	0.60***	0.61***	0.60***	0.58***	0.60***	0.60***	0.60***
Wave 2011 (2002)	0.30***	0.31***	0.30***	0.29***	0.31***	0.31***	0.31***
Df	7	13	13	11	11	21	27
Wald test χ2 value	2423.6***	2436/3***	2522.4***	2921.7***	3031.3***	3377.1***	3388.6***
Wald test χ2 value fort models vs. Model I ^a^	–	95.1***	54.1***	258.9***	349.1***	508.6***	538.4***
Wald test χ2 value for Model VII vs. models ^b^	538.4***	433.5***	477.8***	274.6***	159.8***	34.7***	–

Note: (1) Reference group indicated in parentheses. (2) a, A Wald test for a given model whether the inclusion of new variables significantly improve the goodness of fit compared to Model I; (3) b, A Wald test for Model VII compared to each of other models. (4) + *p* < 0.1, *p < 0.05, **p < 0.01, ***p < 0.001

Table 3 demonstrates that the urban-rural disparity in mortality is generally consistent by age group and gender. Likewise, we found that the urban-rural mortality differential was largely eliminated after including socioeconomic factors but not family/social support, health behaviors, and health-related factors. However, we found that urban older adults still had significantly lower risk of mortality at the oldest-old ages (HR = 0.93; *p* < .05) compared with rural older adults despite inclusion of all factors.

**Table 3:** Adjusted Hazard Ratios of Mortality for Urban-Rural Residence by Age Group and Sex, CLHLS 2002–2014

	Model I	Model II	Model III	Model IV	Model V	Model VI	Model VII
Ages 65–79	0.88**	0.97	0.88**	0.90*	0.90*	0.92+	0.96
Ages 80+	0.91**	0.96	0.90**	0.93*	0.90**	0.91**	0.93*
Women	0.91*	0.98	0.91*	0.91+	0.92+	0.92+	0.98
Men	0.87**	0.96	0.88**	0.91+	0.88**	0.92+	0.96

Note: Model I included demographic background and survey year; Model II added socioeconomic factors to Model I; Model III added family/social support to Model I; Model IV added health behaviors to Model I; Model V added health conditions to Model I; Model VI added family/social support, health behaviors, and health conditions to Model I; Model VII included all study factors

+*p* < 0.1, *p < 0.05, **p < 0.01, ***p < 0.001

## Discussion

This study provides new evidence to understand the urban-rural disparity in mortality among older adults in China. Using longitudinal data from the largest nationally representative study of older adults in the contemporary China, we found that older adults living in urban areas had lower risk of mortality compared with older adults living in rural areas, even at the oldest-old ages, and regardless of gender. We further found that the urban advantage in mortality was largely explained by better socioeconomic factors in urban areas for both women and men and for both young-old and oldest-old adults. On the other hand, we found that factors such as family/social support, health behaviors, and health-related conditions had a limited role in explaining the urban-rural disparity in mortality. These findings support the argument that urban areas in China generally provide greater access to healthcare services and more socioeconomic resources than rural areas [8, 15, 17, 19]. Thus, the positive association we found between living in urban areas and reduced mortality risks was attenuated once individual-level socioeconomic factors were taken into account. This finding is consistent with a previous study conducted in Beijing, which showed that the urban advantage in older-age mortality was either largely reduced or eliminated once individual demographics and socioeconomic characteristics were taken into account [19]. Our findings build on this research and provide new evidence at a national level to highlight the importance of socioeconomic factors in influencing mortality at older ages in a country, such as China.

Overall, our findings of significant urban-rural disparity in mortality at older or oldest ages and between women and men support the persistent inequality theory that the urban-rural disparity in mortality is large and largely unchanged in later life [33, 34]. We found no or little evidence to support the age-as-leveler theory (i.e., diminishing urban-rural disparity in mortality at older and oldest-ages). There is evidence to show that urban-rural difference in mortality seems greater at oldest-old ages as compared to that at young-old ages, which may support the accumulative dis/advantage theory. The more important finding of this study is that urban-rural disparity in mortality at older ages disappeared after including individual socioeconomic factors. This provides empirical support to the materialism and structuralism theory; that is, it is the disparities in socioeconomic resources between urban and rural older adults rather than the disparities in their behaviors that are dominant factors in causing differences in health outcomes [29, 30]. However, there is also some evidence that urban-rural mortality disparity cannot be entirely explained by socioeconomic resources or other factors used in the study at oldest-old ages, which may be because of selective mortality or unobserved heterogeneity. More research is clearly warranted to further disentangle the causes.

Our findings are consistent with previous studies that have shown a mortality advantage among urban older adults relative to rural older adults in China [5, 7, 8, 16]. With few exceptions, however, most of these studies did not distinguish urban-rural mortality differences by age or gender; and none of these studies explored the factors contributing to the differences in mortality by age or gender [5, 7, 16].

The possible reasons for these underlying associations are fourfold. First, older adults with higher SES tend to have a better quality of life in terms of better housing, better neighborhood/residential environments, and better access to facilities for exercise and healthcare [8, 45]. Second, older adults with higher SES have greater access to services that allow them to get timely medical treatment when needed and to benefit from other social services that provide assistance in times of need and/or adversity [46]. Because rural older adults possess a lower standing in society because of their limited resources, they are often socially disadvantaged, marginalized, and even discriminated against, which in turn, create barriers for them to get needed assistance to prevent premature mortality. Third, older adults with higher SES tend to have a better psychological well-being. For example, older adults with greater economic resources are less likely to suffer from financial strains and associated stressors [47]. Relatedly, older adults with higher SES also tend to be more optimistic and have positive attitudes, views, expectations, and perceptions toward their future; and likewise, have more opportunities for involvement in social activities [40]. Finally, people with higher SES are more likely to be aware of and afford healthier diets and lifestyles [45, 48]. All these advantages/privileges possessed by socioeconomic resourceful older adults could offset the contextual or institutional disadvantages (such as living in a rural area) to their health.

A key strength of the present study is the use of a large national sample, which included more than 28,000 older adults, to examine urban-rural disparity in mortality over a 12-year period. The large sample and repeated longitudinal measures used in this study allowed us to obtain robust results of the potential factors associated with urban-rural differences in mortality in men and women and across age groups [8]. Another strength of this study is that the data come from a nationally representative survey of older adults in China, a transitional non-Western society, where there are established institutions in place for socioeconomic development, health care, and pension systems. Such institutional differences could result in unintended disparities in the resources available to individuals for their health promotion. A third uniqueness of the present study is the comparison of urban-rural mortality disparity between men and women and between adults at ages 65–79 and ages 80+. As noted earlier, we build upon previous studies by providing new evidence of urban-rural disparity among key subpopulations in a developing country such as China.

### Policy implications

Our findings have important implications for public policy and planning. First, in contemporary China, rural older adults face significant social disadvantages compared to their urban counterparts. Older adults in rural areas have much less access to healthcare and lower pension benefits because of the urban-rural dual development system. Therefore, reforms to improve these institutional systems and formulate favorable policies that increase rural older adults’ access/benefits to public goods such as healthcare and pensions are clearly warranted [4, 7]. Relatedly, rural development should be highlighted in the national socioeconomic strategic plans. Intervention programs focusing on socioeconomic development and poverty reduction in rural areas should be prioritized. These macro-perspective policies are indeed the necessary foundation to narrow the urban-rural differences in healthcare and social services, improved housing and infrastructure, and overall better economic conditions. To have equal access to healthcare and adequate social security is human right that is consistent with SDG guidelines. Thus, only when there is greater equality/equity between urban and rural residents in their access to healthcare and pension systems can rural residents achieve similarly low risks of mortality as urban residents.

Second, the State (or the local rural communities) should develop free or affordable healthcare or social services delivered though home visits to rural older adults. With limited resources, rural older adults often cannot afford or access these services, which in turn, contribute to worsening health and increased mortality risks. According to the CLHLS, 53 and 12% of rural older adults attributed their reasons of not seeking medical treatment when in needed to limited financial resources and difficulties in access healthcare, respectively, compared with 46 and 5% among urban older adults. Such home- and community-based services could indirectly improve their access to resources and help rural older adults receive regular health check-ups and prevention services that would otherwise not be affordable or accessible. These programs also could improve older adults’ knowledge about the benefits of a healthy lifestyle via dissemination of health literacy information. Indeed, there is existing evidence to show that favorable healthcare policies and programs may mitigate health disparity in later life [49].

### Limitations

Several limitations should be taken into account when interpreting our findings. First, our study only used information on current residence and did not consider possible changes in urban-rural residence during an individual’s life course. Studies have shown an association between urban and rural exposure during his/her lifetime and health-related outcomes at later ages [4, 39, 50]. Relatedly, although our classification of urban-rural residence is consistent with official definitions used by the Chinese government, we were not able to determine whether possible rural-to-urban residence change was permanent (obtained an urban *hukou* status) or temporary (living in urban areas with rural *hukou*). Alternatively, with rapid urbanization and in situ urbanization, the residential status of many rural inhabitants may have changed even if their current residence remained in the same location, village, or township [51]. Therefore, we recognize that urban-rural residence may be dynamic over one’s lifetime and incorporating such information may provide additional insights into urban-rural mortality differentials [4].

Second, we also recognize that the urban-rural health disparity is also directly or indirectly influenced by many biological, environmental, and/or other contextual factors as embedded in the BPS framework. Unfortunately, a lack of these measures in the CLHLS prohibited us from examining them in the current study. With regard to contextual factors, urban areas often have better infrastructure (e.g., transportation and safe drinking-water) than rural areas—particularly in China with the implementation of the urban-rural dual development system starting in the 1950s—which certainly contributes to excessive mortality in rural areas [8]. We also did not consider increasing crowdedness and polluted environments—thus, urban residents may have greater exposure to environments with relatively greater health-risks than their rural peers [52]. In addition to further including potential biological factors in the analyses, it is important for future research to consider the environmental, socio-political, and other contextual factors characterizing the living environments of older adults in urban and rural areas [17, 22].

Third, we acknowledge that health/mortality selection may affect our findings. This selection process has two aspects. On the one hand, the rural population may exhibit a loss of “healthier people” due to their out-migration to urban areas. According to some research, older adults who migrated from rural-to-urban areas when they were young have lower risk of mortality compared with rural older adults who did not migrate [4]. Because these migrants are generally healthier and more socioeconomically advantaged than rural non-migrant counterparts [4, 53], it is possible that such migration could make the current urban population healthier because of the commensurate changes in population composition [4]. On the other hand, older adults in rural China likely encountered greater adversities in their life—and consequently had higher rates of mortality in earlier life—which may result in healthier older adults as their rural frail peers were eliminated from the cohort [4, 54].

Finally, as noted earlier, the CLHLS did not include nine minority provinces where urbanization, socioeconomic development, and healthcare coverage are relatively low in comparison with the other 22 sampled provinces. Consequently, mortality rates at older ages in these nine provinces are higher than those of the 22 sampled provinces [55]. However, it is unclear whether the urban-rural difference in these non-included provinces is more or less pronounced compared with the sampled provinces. Fortunately, because the overall proportion of the population from these non-sampled provinces is relatively small (around 10%), we remain confident about the robustness of our findings. Nevertheless, we encourage additional studies to include these nine provinces to further verify our findings.

## Conclusions

By using five waves of a nationally representative survey with large sample of nearly 30,000 older adults in China, we investigated the role of socioeconomic conditions, family/social support, health behaviors, and baseline health status in contributing to urban-rural differences in mortality at older ages. Our study found that differences in socioeconomic factors between urban and rural areas were the primary causes for the urban-rural disparity in mortality at older ages, and that other factors such as family/social support, health behaviors, and health-related factors had a limited influence on the urban-rural mortality disparity. These conclusions were generally consistent by age group and gender. Our findings have important implications for possibly intervention programs aiming to close the urban-rural mortality gap at older ages.

## Supplementary information

**Additional file 1 Appendix Table S1.** Adjusted Odds Ratios (OR) for Loss to Follow-up and Relative Hazard Ratios (HR) for Mortality with Imputation, CLHLS, 2002–2014.

## Data Availability

The CLHLS datasets are publicly available at the National Archive of Computerized Data on Aging, University of Michigan (http://www.icpsr.umich.edu/icpsrweb/NACDA/studies/36179). Researchers can obtain these data after submitting a data use agreement to the CLHLS team.

## Abbreviations

ADL: Activities of daily living
CLHLS: Chinese Longitudinal Healthy Longevity Survey
HR: Hazard ratios
IADL: Instrumental activities of daily living
MMSE: Mini-mental State Examination
NBSC: National Bureau of Statistics of China
