# Supplementary material for: Individual-level factors attributable to urban-rural disparity in mortality among older adults in China
Source: BMC Public Health. 2020 Sep 29;20:1472. doi: 10.1186/s12889-020-09574-9 (PMC7526413; doi:10.1186/s12889-020-09574-9)
Supplement: Supplementary file 1 — Additional file 1 Appendix Table S1. Adjusted Odds Ratios (OR) for Loss to Follow-up and Relative Hazard Ratios (HR) for Mortality with Imputation, CLHLS, 2002–2014. [file 12889_2020_9574_MOESM1_ESM.pdf]

Appendix

Table A. Adjusted Odds Ratios (OR) for Loss to Follow-up and Relative Hazard Ratios (HR) for Mortality with Imputation, CLHLS, 2002-2014

	ORs for Loss to Follow-up	Relative HRs for Mortality [a]						
		Model I	Model II	Model III	Model IV	Model V	Model VI	Model VII
Urban (rural)	1.81***	0.91**	1.01	0.91**	0.93+	0.91**	0.94+	0.98
**Demographic Background**								
Age	0.99**	1.11***	1.10***	1.10***	1.09***	1.08***	1.07***	1.06***
Male	1.00	1.35***	1.50***	1.43***	1.45***	1.56***	1.63***	1.67***
Han (non-Han)	2.06***	0.95	0.97	0.95	0.95	0.87+	0.88+	0.89+
**Socioeconomic Factors**								
1-6 years of schooling (0)	0.98		0.95					1.04
7+ years of schooling (0)	1.095		0.83**					0.94
Economic independence (no)	1.069		0.71***					0.81***
White collar occupation (no)	1.05		1.09+					1.04
Good family economic condition (no)	0.99		0.93					1.03
Adequate access to healthcare (no)	1.08		0.77***					0.91+
**Family/Social Support**								
Currently married (no)	0.80***			0.78***			0.82**	0.84**
Close proximity to children (no)	0.66***			1.07			1.04	1.02
Primary support, child/relative (spouse)	0.95			0.95			0.94	0.92
Primary support, friend (spouse)	0.92			0.82**			0.87*	0.86*
Primary support, other (spouse)	1.17			1.46**			1.13	1.12
Primary support, nobody (spouse)	0.86+			1.59***			1.12	1.10
**Health Behaviors**								
Leisure activity level, intermediate (low)	0.99				0.46***		0.66***	0.67***
Leisure activity level, high (low)	1.02				0.32***		0.53***	0.55***
Currently smoking (no)	0.90*				0.91*		0.97	0.97
Ever engaged in physical labor (no)	0.74***				0.99		1.02	0.98
**Health Conditions**								
IADL disabled (no)	1.13**					1.69***	1.55***	1.52***
ADL disabled (no)	1.18***					1.94***	1.65***	1.66***
Cognitively impaired (no)	0.92*					1.56***	1.38***	1.37***
Has 1+ chronic disease (no)	0.99					1.11**	1.12**	1.13***
**Survey Interval**								
Waves 2005-2008 (2002-2005)	1.79***	0.88**	0.89**	0.86**	0.88**	0.90*	0.88**	0.89**
Waves 2008-2011 (2002-2005)	1.70***	0.78***	0.80***	0.78***	0.78***	0.85***	0.82***	0.83***
Wave 2011-2014 (2002-2005)	1.81***	0.70***	0.70***	0.69***	0.70***	0.70***	0.69***	0.69***
df	27	7	13	13	11	11	21	27
Wald $\chi2$	1290.8***	2454.0***	2440.2***	2687.3***	3503.1***	3625.2***	3990.0***	3948.0***
Wald $\chi2$ for models vs. Model I [b]	---	---	113.2***	137.7***	518.9***	723.5***	885.7***	937.6***
Wald $\chi2$ for Model VII vs. models [c]	---	937.6***	786.1***	800.7***	527.5***	232.2***	23.74***	---

Note: (1) Reference group indicated in parentheses. (2) a, The relative hazard ratios for mortality were obtained from imputed data for those who were lost to follow-up. (3) b, Wald $\chi2$ tests for a given model to assess whether the inclusion of new variables significantly improved the goodness of fit compared to Model I; (4) c, Wald $\chi2$ tests for Model VII compared to each of the other models. (5) +p<0.1, *p<0.05, **p<0.01, ***p<0.001.
